# Annexin A6 mitigates neurological deficit in ischemia/reperfusion injury by promoting synaptic plasticity

**DOI:** 10.1111/cns.14639

**Published:** 2024-02-21

**Authors:** Yilin Wang, Zhenhong Yang, Rongliang Wang, Yangmin Zheng, Ziping Han, Junfen Fan, Feng Yan, Ping Liu, Yumin Luo

**Affiliations:** ^1^ Institute of Cerebrovascular Disease Research and Department of Neurology Xuanwu Hospital of Capital Medical University Beijing China; ^2^ Beijing Institute for Brain Disorders Beijing China

**Keywords:** ischemia, membrane, neuron, plasticity, repairment, synapse

## Abstract

**Aims:**

Alleviating neurological dysfunction caused by acute ischemic stroke (AIS) remains intractable. Given Annexin A6 (ANXA6)'s potential in promoting axon branching and repairing cell membranes, the study aimed to explore ANXA6's potential in alleviating AIS‐induced neurological dysfunction.

**Methods:**

A mouse middle cerebral artery occlusion model was established. Brain and plasma ANXA6 levels were detected at different timepoints post ischemia/reperfusion (I/R). We overexpressed and down‐regulated brain ANXA6 and evaluated infarction volume, neurological function, and synaptic plasticity‐related proteins post I/R. Plasma ANXA6 levels were measured in patients with AIS and healthy controls, investigating ANXA6 expression's clinical significance.

**Results:**

Brain ANXA6 levels initially decreased, gradually returning to normal post I/R; plasma ANXA6 levels showed an opposite trend. ANXA6 overexpression significantly decreased the modified neurological severity score (*p* = 0.0109) 1 day post I/R and the infarction area at 1 day (*p* = 0.0008) and 7 day (*p* = 0.0013) post I/R, and vice versa. ANXA6 positively influenced synaptic plasticity, upregulating synaptophysin (*p* = 0.006), myelin basic protein (*p* = 0.010), neuroligin (*p* = 0.078), and tropomyosin‐related kinase B (*p* = 0.150). Plasma ANXA6 levels were higher in patients with AIS (1.969 [1.228–3.086]) compared to healthy controls (1.249 [0.757–2.226]) (*p* < 0.001), that served as an independent risk factor for poor AIS outcomes (2.120 [1.563–3.023], *p* < 0.001).

**Conclusions:**

This study is the first to suggest that ANXA6 enhances synaptic plasticity and protects against transient cerebral ischemia.

## Graphical abstract

After acute ischemic stroke, ANXA6 transfers from cytoplasm to cell membrane, exerting a protective role. ANXA6 overexpression can increase expression levels of MBP, TRKB, neuroligin, and synaptophysin, alleviating neurological deficits.

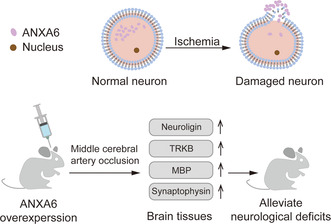

## 1 INTRODUCTION

Worldwide, stroke is the second leading cause of mortality and the primary cause of disability.^1^ Acute ischemic stroke (AIS), a main subtype, refers to a rapid reduction of blood supply to the brain, resulting in impaired brain function.^2^ Currently, the two widely accepted therapies for AIS are intravenous thrombolysis using recombinant tissue plasminogen activator (alteplase) and blood clot removal (thrombectomy).^2^, ^3^ However, these therapies are limited to the therapeutic time window and the therapeutic effects are not effective for all the patients. Therefore, alleviating neurological function damage due to ischemic stroke remains a major challenge.

The annexin family, comprising 12 members, belongs to Ca^2+^‐dependent phospholipid‐binding proteins.^4^ Annexin A6 (ANXA6), with the largest molecular weight and most annexin cores, contributes to membrane transport and cellular signal conduction.^5^ ANXA6 has been extensively observed in neurons and vascular smooth muscle cells, among others. ANXA6 is mainly located in dendrites, cell membranes, and nervous fibers in pyramidal neurons.^6^ During primary neuron development, ANXA6 accumulates at the initial sites of axons, significantly promoting axon branches.^7^ This effect is possibly owing to ANXA6 regulating membrane–actin interactions in axons and nerve terminals.^8^ Additionally, cell membrane damage can lead to various diseases. ANXA6 forms a repair cap at lesion sites, promoting cell membrane repair. Notably, ANXA6‐mediated repair caps have been found in impaired neurons.^9^ Despite ischemia causing neuron injury, which induces neurological dysfunction, the role of ANXA6 in AIS remains unreported.

## 2 METHODS

### 2.1 Animal care

Our animal experiments adhered to ethical requirements and were approved by the Institutional Animal Care and Use Committee of Capital Medical University (XW‐20220829‐2). Adult male C57/BL6 mice (8–10‐weeks old) weighing 20–25 g were obtained from Vital River Laboratory Animal Technology Co., Ltd. (Beijing, China). The mice were reared under standardized conditions.

### 2.2 Experimental protocols

#### 2.2.1 Experiment I

To explore ANXA6 expression levels in the brain and plasma post AIS, we randomly categorized the mice into six groups based on whether they experienced middle cerebral artery occlusion (MCAO) and reperfusion injury and the time of brain tissue and plasma collection: sham, 6 h, 1 day, 3 days, 7 days, and 28 days post ischemia/reperfusion (I/R) (*n* = 5) (Table S1).

#### 2.2.2 Experiment II

To determine whether ANXA6 can improve AIS‐induced neurological dysfunction, we used an intracerebroventricular injection (ICV) of lentivirus to intervene in ANXA6 expression in the brain. We decreased ANXA6 levels in the brain using the ICV of ANXA6 shRNA lentivirus (sh ANXA6), and a control group was established using the ICV of a nonsensical oligonucleotide control (NC). Additionally, we increased ANXA6 levels in the brain using the ICV of a lentiviral vector overexpressing ANXA6 (ANXA6 OE), and a control group was established using the ICV of an empty vector (EV). Briefly, 30 mice were randomly categorized into five groups: sham, MCAO + sh ANXA6, MCAO + NC, MCAO + EV, and MCAO + ANXA6 OE (*n* = 6) (Table S1). Excluding those in the sham group, all mice were subjected to MCAO 7 days after the lentivirus ICV, and brain tissues and plasma were collected 7 days post I/R. Cerebral infarction volume, brain edema, body weight, Longa, and Modified neurological severity score (mNSS) scores were evaluated 1 day and 7 day post I/R to assess the role of ANXA6 in the brain post I/R.

### 2.3 Transient focal cerebral ischemia and neurological evaluation

A transient focal cerebral ischemia was induced in C57/BL6 mice through transient MCAO,^10^ followed by reperfusion after 60 min. Mice were anesthetized with 3.5% enflurane in N_2_O:O_2_ (70%:30%). During surgery, vital signs (e.g., mean arterial blood pressure and heart rate) were kept stable, and rectal temperature was consistently maintained at 37 ± 0.5°C. Neurological function was evaluated using the Longa and mNSS scoring systems by a blinded observer 1 day and 7 days post reperfusion.

### 2.4 Intracerebroventricular injection of lentivirus

The lentiviruses used in this study were purchased from Hanbio Tech (Shanghai, China). We overexpressed ANXA6 and down‐regulated ANXA6 in the brain using the lentivirus ICV. Lentiviruses encoding shRNAs for ANXA6 were defined as sh ANXA6, and a nonsensical oligonucleotide served as the control. The lentiviral vector overexpressing ANXA6 was defined as ANXA6 OE, and an empty vector served as the control. The lentivirus ICV was conducted based on procedures described by Luo et al.^11^ Approximately 7 days after lentivirus injection, the mice were subjected to MCAO.

### 2.5 Calculation of infarction volume

Mouse brain tissues were collected, stained with 2,3,5‐triphenyl tetrazolium chloride (TTC), sliced, and photographed. Images were analyzed using ImageJ software (National Institute of Health, Bethesda, MD, USA).^12^ Subsequently, the infarction volume was calculated using the following equation: $c=\left(a-b\right)÷a\times 100\%$, where *a* is the contralateral entire area, *b* is the ipsilateral non‐infarction area, and *c* is the relative infarction volume (%).^13^

### 2.6 Western blot analysis

Infarcted hemispheres were homogenized and lysed using RIPA lysis buffer (G2002, Servicebio, Wuhan, China), supplemented with protease inhibitor cocktail (Roche) and phosphatase inhibitor cocktail (Roche), followed by sonication on ice. The supernatant was collected after centrifugation (12,000 *g*, 30 min). Protein concentration was quantified using a BCA reagent (G2026, Servicebio, Wuhan, China). Western blot procedures were based on previous literature.^14^ The primary antibodies used in our study comprised the following: anti‐annexin A6 rabbit polyclonal antibody (1:2000 dilution, 12,542‐1‐AP, Proteintech), anti‐neuroligin rabbit polyclonal antibody (1:1000 dilution, ab36602, Abcam), anti‐myelin basic protein (MBP) rabbit polyclonal antibody (1:1000 dilution, ab40390, Abcam), anti‐TrkB rabbit polyclonal antibody (1:1000 dilution, ab18987, Abcam), anti‐synaptophysin rabbit monoclonal antibody (1:5000 dilution, ab52636, Abcam), and anti‐β‐actin mouse monoclonal antibody (1:2000 dilution, GB15001‐100, Servicebio). The appropriate secondary antibodies used were horseradish peroxidase‐conjugated goat anti‐rabbit (1:2500 dilution, ZB‐2301, ZSGB‐BIO, Beijing, China) or goat anti‐mouse antibody (1:2500 dilution, ZB‐2305, ZSGB‐BIO, Beijing, China).

### 2.7 Immunofluorescence staining

Mouse brain tissues were coronally sliced into 10‐μm thick sections and subjected to immunofluorescence staining as described previously.^15^ Primary antibodies included anti‐annexin A6 rabbit polyclonal antibody (1:50 dilution, 12,542‐1‐AP, Proteintech), anti‐annexin A6 mouse monoclonal antibody (1:50 dilution, sc‐271,859, Santa Cruz Biotechnology), and antineuronal nuclei mouse monoclonal antibody (1:50 dilution, MAB377, EMD Millipore Corporation). Appropriate fluorescent‐labeled secondary antibodies were used.

### 2.8 Study population

Our study adhered to the Declaration of Helsinki and was approved by the Ethics Committee of Xuanwu Hospital, Capital Medical University ([2021]079). Written informed consent was waived. Patients who were admitted to the stroke unit between November 2018 and July 2019 at our hospital and diagnosed with AIS based on the criteria published in 2011^16^ were enrolled. The exclusion criteria were as follows: (1) history of hepatic or renal dysfunction, (2) history of other neurological diseases, (3) long‐term medication history, (4) neurological dysfunction before AIS onset, (5) hematological or organic diseases, and (6) tumors.

### 2.9 Clinical variables

The baseline characteristics of the patients were reviewed, including sex, age, laboratory results, medical history, infarction volume, National Institute of Health Stroke Scale (NIHSS) score, modified Rankin scale (mRS) score at 3 months, and treatment regimen (administration of rtPA or mechanical thrombectomy). Laboratory test results were classified as “normal” or “abnormal” based on reference intervals. AIS severity was stratified into four levels according to NIHSS scores: slight (≤4), moderate (5–15), moderate to severe (16–20), and severe (≥21). The AIS prognosis was classified into favorable (mRS ≤2) and poor (mRS >2 and death cases) outcomes based on mRS scores.

### 2.10 Enzyme‐linked immunosorbent assay

Approximately 500 μL of blood was collected from the inferior vena cava of each mouse. Approximately 3 mL of peripheral blood was collected from each patient and the healthy control group upon admission before therapeutic intervention. Subsequently, the patient and mouse blood samples were processed to extract plasma. Plasma ANXA6 levels were assessed using enzyme‐linked immunosorbent assay (ELISA) kits (EH1625, FineTest, Wuhan, China).

### 2.11 Statistical analysis and graph plotting

Patient baseline characteristics are presented as means ± SD or medians, with interquartile ranges based on continuous or categorical variables, respectively. Categorical variables were analyzed using Chi‐square test or Fisher exact test. Regarding continuous variables, all data distribution normalities were assessed through Shapiro–Wilk and Kolmogorov–Smirnov tests using GraphPad Prism 8.0 (GraphPad Software Inc., San Diego, California, United States). Data with normal distribution were analyzed using the Student's *t*‐test. The data did not conform to a normal distribution; however, the Mann–Whitney *U* test was used to analyze the data. Statistical analysis was conducted using IBM SPSS statistics version 23.0 (IBM Corp., Armonk, NY), and a *p‐*value <0.05 was considered statistically significant. The logistic regression model was established through a backward‐forward selection procedure using R software (Version 4.4.1, R Foundation for Statistical Computing, Vienna, Austria). The figures were generated using GraphPad Prism 8.0 (GraphPad Software Inc.) and R software Version 4.4.1 (R Foundation for Statistical Computing).

## 3 RESULTS

### 3.1 Cerebral and plasma ANXA6 responses to transient cerebral I/R

**FIGURE 1:**
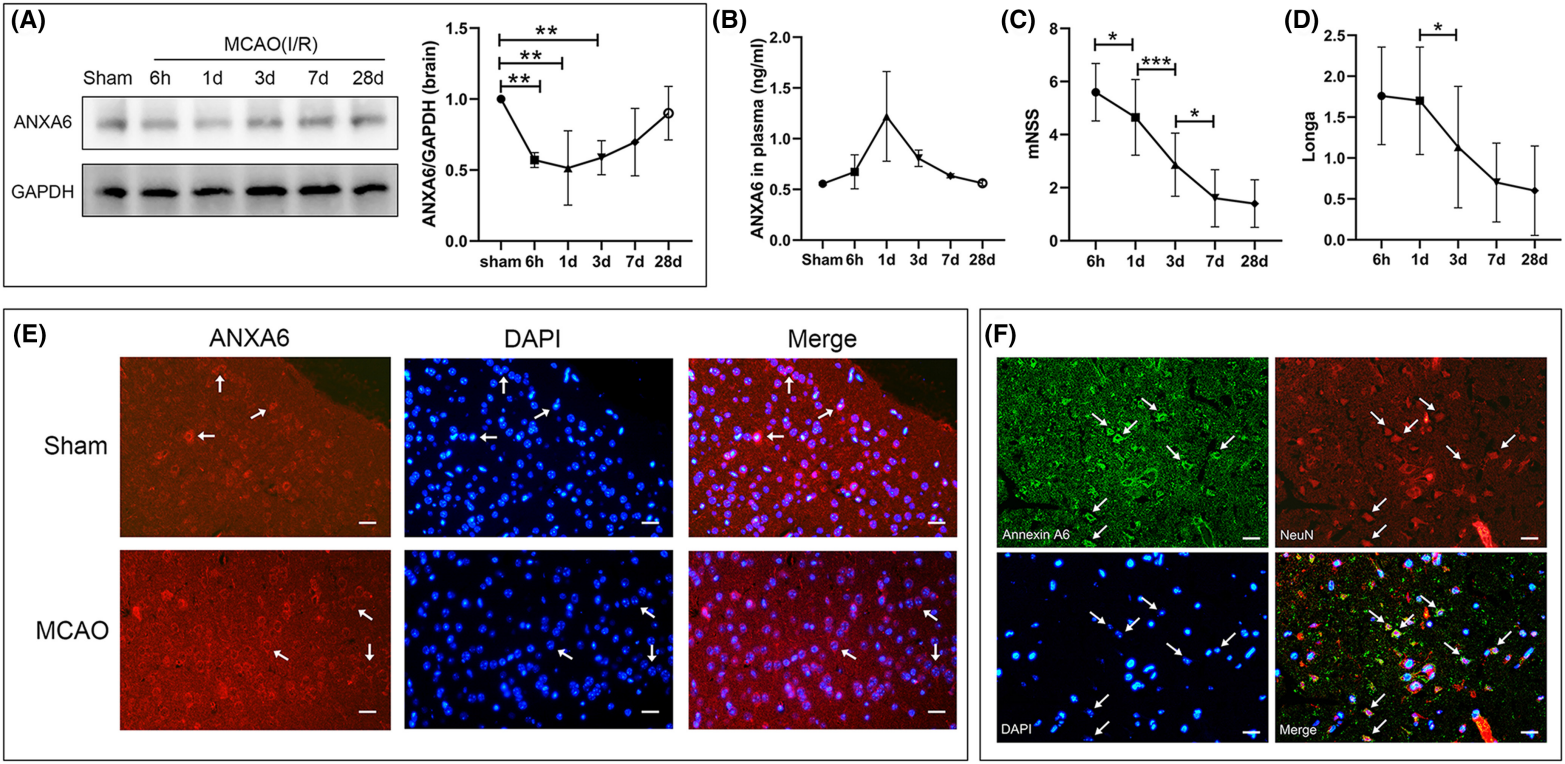
Cerebral and plasma ANXA6 level response to transient cerebral ischemia/reperfusion. (A) Western blotting results of ANXA6 level in the brain at different timepoints after I/R. MCAO: middle cerebral artery occlusion. I/R: ischemia/reperfusion. (B) Enzyme‐linked immunosorbent assay results of plasma ANXA6 level at different timepoints after I/R. (C) and (D) Longa and mNSS score results at different timepoints after I/R. *p < 0.05; **p < 0.01; ***p < 0.001. (E) Immunofluorescence staining for ANXA6 receptor (red) and DAPI (blue) in brain sections (sham and MCAO at day 7 after I/R) (arrowheads), Scale bars = 20 μm. (F) Immunofluorescence staining for ANXA6 receptor (green), NeuN (neuronal marker, red) (arrowheads), and DAPI (blue) in brain sections. The ANXA6 colocalized in the neurons (arrowheads) on day 7 post I/R. Scale bars = 20 μm.

We initially explored the variation trend in ANXA6 expression levels in the brain and plasma at 6 h, 1 day, 3 days, 7 days, and 28 days post I/R using western blotting and ELISA. Western blotting results showed an initial decrease in brain ANXA6 levels post I/R, followed by a gradual increase to normal levels approximately 28 days post I/R (Figure 1A). The differences between the sham group and the 6 h, 1 day, and 3 days post I/R groups were statistically significant (*p* = 0.005). Figure 1B shows an increase in plasma ANXA6 levels post I/R and a subsequent decrease to normal levels 28 days post I/R, contrary to brain levels. Figure 1C,D demonstrate a gradual decrease in Longa and mNSS scores from 6 h to 28 days post I/R. Additionally, Figure 1E displays that ANXA6 was extensively distributed in the cytoplasm under normal conditions. However, post I/R, ANXA6 accumulated mostly on the cell membrane. Figure 1F shows the colocalization of ANXA6 and NeuN post I/R.

### 3.2 ANXA6 alleviates I/R‐induced brain injury

To explore the role of ANXA6 in cerebral I/R injury, we established ANXA6 OE, EV, sh ANXA6, NC, and sham groups. The cerebral infarction volume, brain edema, body weight, Longa, and mNSS scores were evaluated 1 day and 7 days post I/R. Additionally, synaptic plasticity‐related factors were detected 7 days post I/R.

#### 3.2.1 Overexpression of ANXA6 protects the brain against I/R injury

**FIGURE 2:**
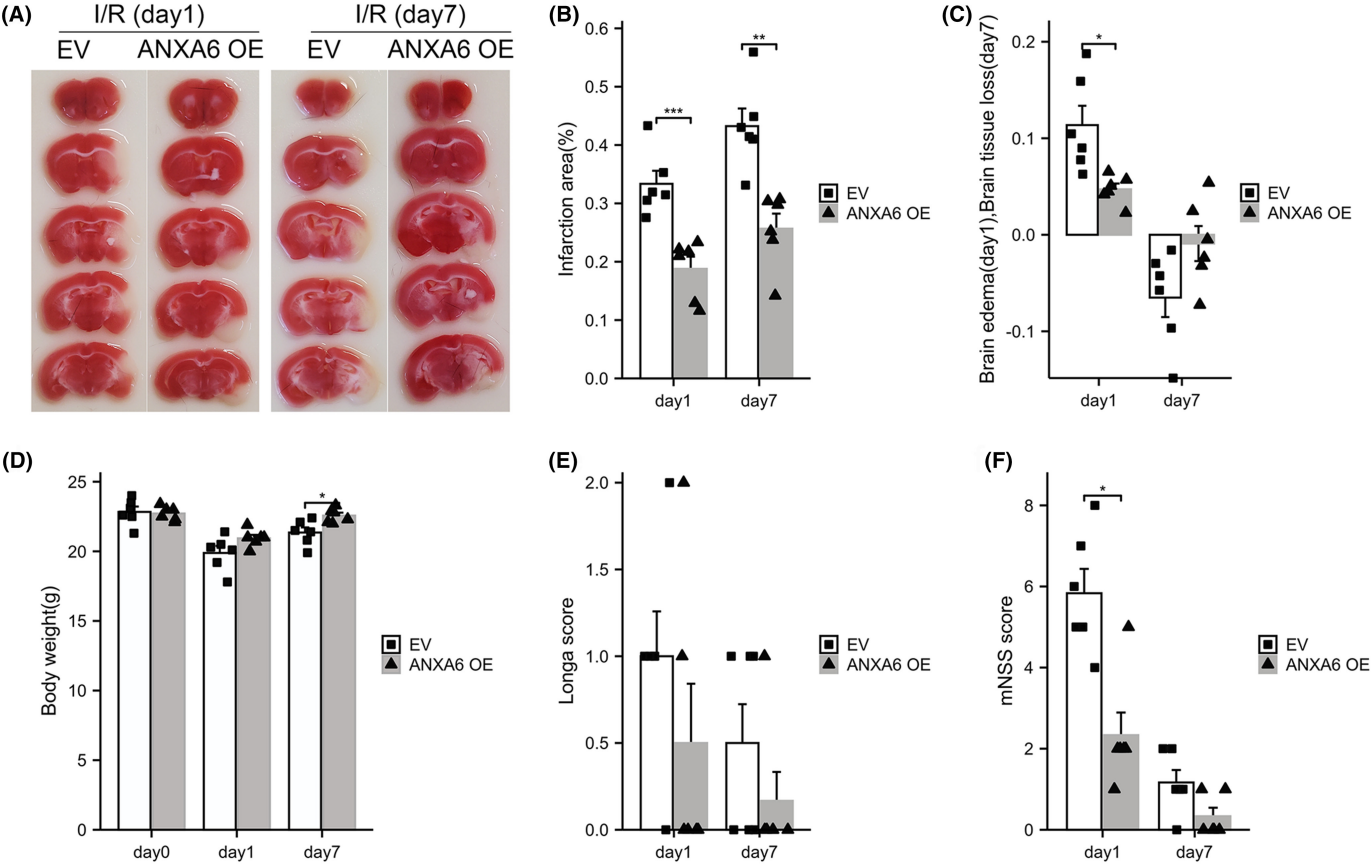
Over‐expression of ANXA6 protects the brain against I/R injury. I/R: ischemia/reperfusion, EV: empty vector, OE: overexpression. (A) 2,3,5‐triphenyltetrazolium chloride staining of brain tissue at day 1 and day 7 after I/R. (B) Quantification of infarct volume at day 1 and day 7 after reperfusion. (C) Quantification of brain edema (day 1) and brain tissue loss (day 7) after reperfusion. (D) Body weight was assessed immediately (day 0), day 1, or day 7 after I/R. (E) and (F) Longa and mNSS scores were assessed on day 1 or day 7 post I/R. *p < 0.05; **p < 0.01; ***p < 0.001.

Figure 2A displays the TTC staining of mouse brain slices. ANXA6 OE significantly decreased the infarction area compared to the EV group at 1 day (*p* = 0.0008) and 7 days (*p* = 0.0013) post I/R (Figure 2B). Figure 2C displays that ANXA6 OE significantly alleviated brain edema 1 day post I/R (*p* = 0.0196). At 7 days post I/R, brain tissue loss in the ANXA6 OE group was less than that in the EV group, with no statistical significance. Regarding body weight, no significant differences were observed between the two groups 1 day post I/R (Figure 2D). However, 7 days after I/R, the body weight of the ANXA6 OE group significantly surpassed that of the EV group (*p* = 0.0168). Figure 2E shows that ANXA6 OE decreased Longa scores 1 day and 7 days post I/R, with no statistical significance. Furthermore, Figure 2F shows that the mNSS scores in the ANXA6 OE group were significantly lower than those in the EV group (*p* = 0.0109) 1 day after I/R. However, no significant differences were observed in the mNSS scores between the two groups 7 days post I/R.

#### 3.2.2 sh ANXA6 exacerbates I/R‐induced brain injury

**FIGURE 3:**
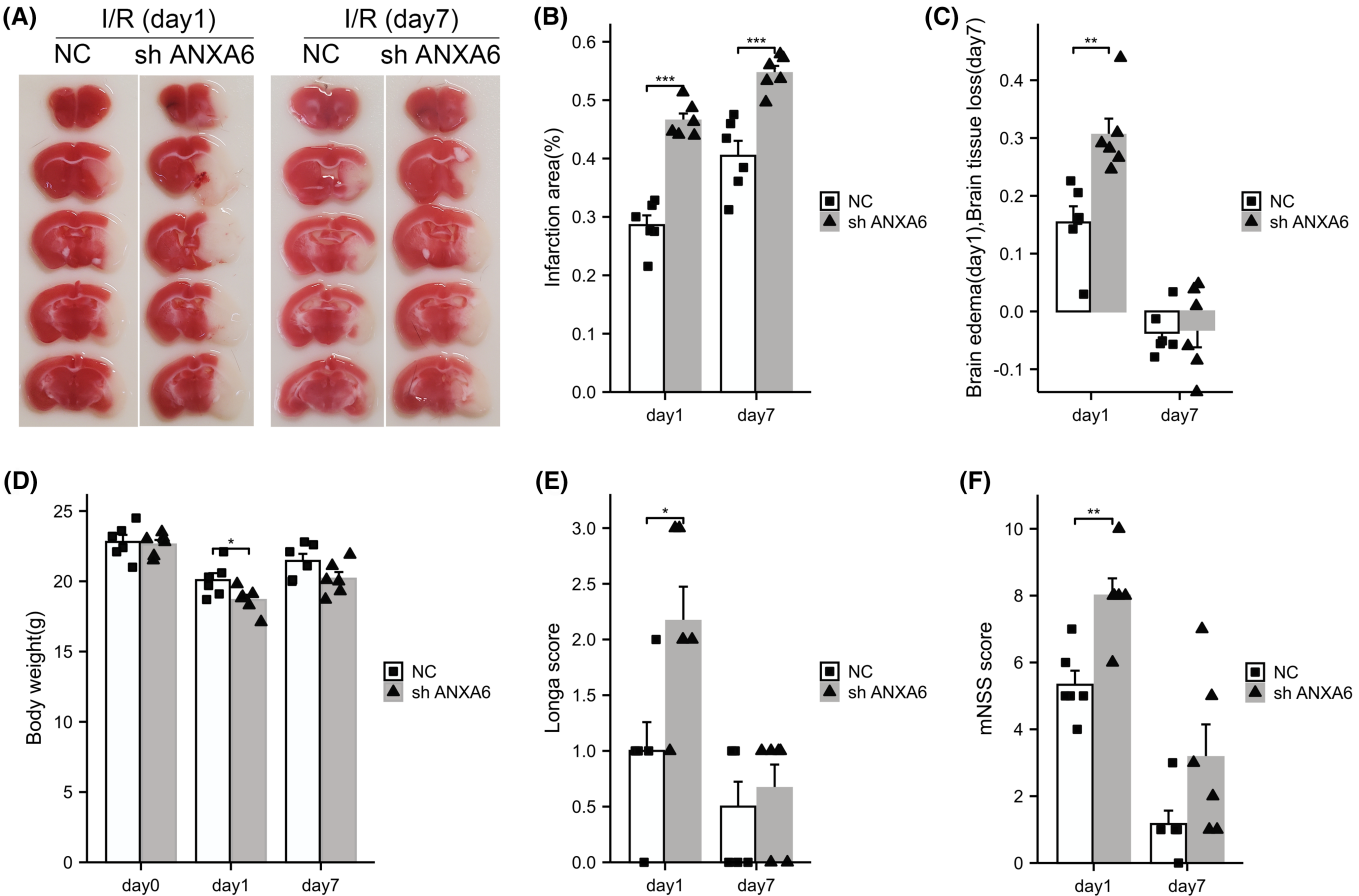
sh ANXA6 exacerbates the brain injury caused by I/R. I/R: ischemia/reperfusion, NC: nonsensical oligonucleotide control. (A) 2,3,5‐triphenyltetrazolium chloride staining of brain tissue at day 1 and day 7 after I/R. (B) Quantification of infarct volume on day 1 and day 7 after reperfusion. (C) Quantification of brain edema (day 1) and brain tissue loss (day 7) after reperfusion. (D) Body weight was assessed immediately (day 0), day 1, or day 7 after I/R. (E) and (F): Longa and mNSS scores were assessed on day 1 or the day 7 after I/R. *p < 0.05; **p < 0.01; ***p < 0.001.

The TTC staining of mouse brain slices is shown in Figure 3A. Figure 3B shows that compared to the NC group, sh ANXA6 increased the infarction area 1 day (*p* < 0.001) and 7 days (*p* < 0.001) after I/R. Figure 3C displays that sh ANXA6 significantly aggravated brain edema 1 day post I/R (*p* = 0.0034). However, 7 days post I/R, neither group observed a significant difference in brain tissue loss. Figure 3D shows that sh ANXA6 significantly aggravated body weight loss 1 day after I/R (*p* = 0.0455), but no significant differences were observed in body weight loss between groups 7 days after I/R. Figure 3E displays that compared to the NC group, sh ANXA6 increased Longa scores by 1 day after I/R (*p* = 0.0275). However, the Longa scores of the sh ANXA6 group were slightly higher than those of the NC group 7 days after I/R, with no statistically significant difference. Figure 3F shows that the mNSS score of the sh ANXA6 group was significantly higher than that of the NC group (*p* = 0.0085) at 1 days post I/R. Furthermore, the mNSS scores of the two groups 7 days after I/R showed a similar tendency to those observed 1 day after I/R, with no statistical significance.

### 3.3 ANXA6 regulates neuroplasticity

Neuroplasticity depends on cytoskeletal rearrangement, membrane morphology, and protein levels.^17^ ANXA6 regulates membrane microdomains and lipid raft arrangement, participating in endocytosis and exocytosis.^18^ Lipid rafts refer to membrane microdomains rich in cholesterol, sphingolipid lipids, and proteins,^19^ participating in the signal transduction of neurons and other cells.^20^ The synaptic vesicle membrane is a cholesterol‐dependent microdomain. Synaptophysin, a transmembrane protein found in synaptic vesicles, interacts with plasma membrane cholesterol, vital for synaptic vesicle endocytosis.^21^ Neuroligin, a synaptic cell‐adhesion molecule and a membrane protein within neurons, typically accumulates on the postsynaptic membrane and is involved in transsynaptic junctions, synapse formation, and synaptic plasticity.^22^, ^23^ MBP, a membrane‐based intrinsically disordered protein, can interact with lipid membranes and link myelin layers surrounding axons, participating in compacting the myelin sheath during neural repair and regeneration after injury.^24^, ^25^ TrkB, typically located on lipid rafts, participates in synaptic transmission and plasticity by interacting with the brain‐derived neurotrophic factor (BDNF). Lipid rafts are vital for BDNF–TrkB signaling.^26^

#### 3.3.1 ANXA6 OE increases the level of synaptic plasticity–related proteins in the brain

**FIGURE 4:**
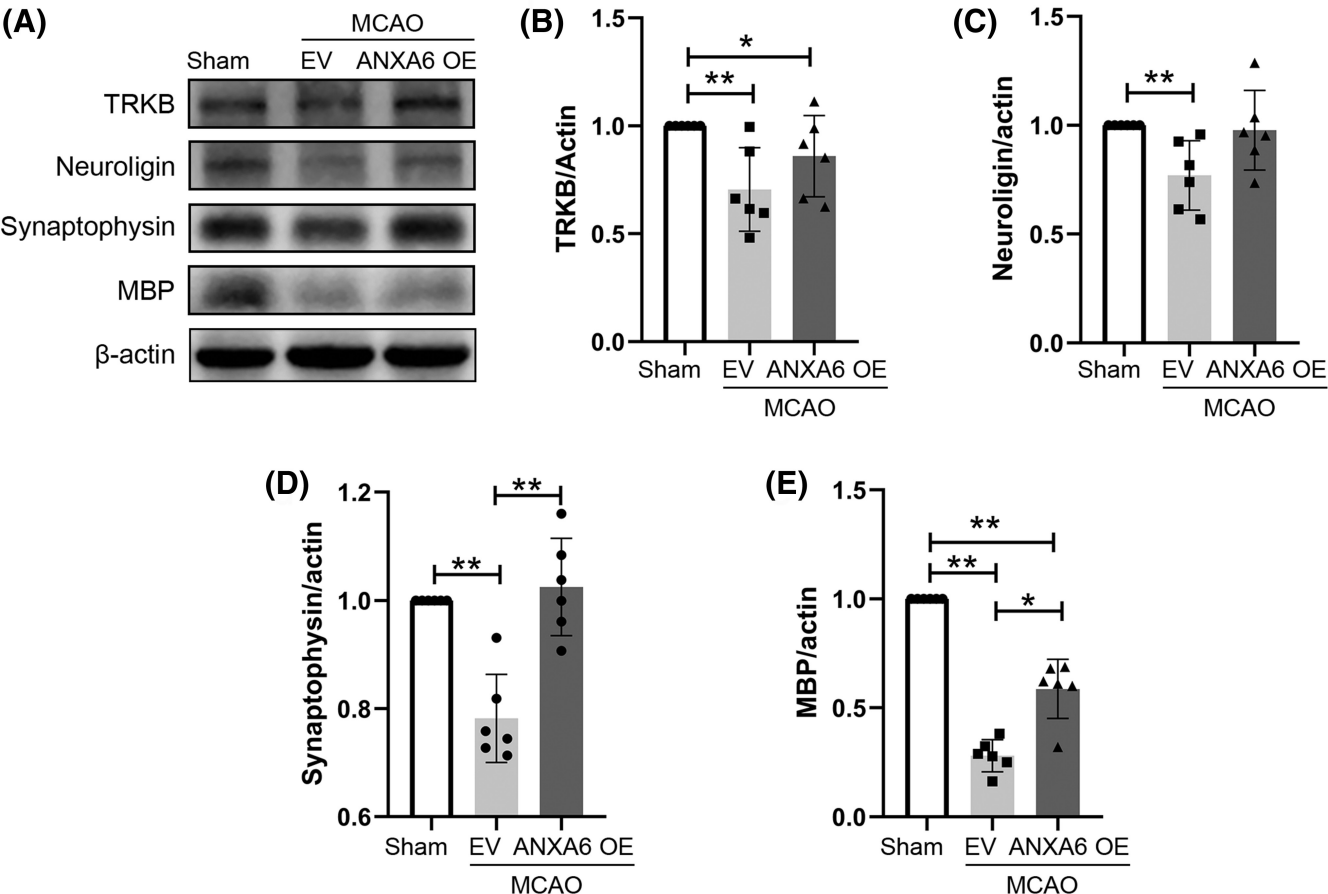
ANXA6 over‐expression increased the synaptic plasticity‐related proteins. MCAO: middle cerebral artery occlusion. TRKB: Tropomyosin‐related kinase B, MBP: Myelin basic protein. EV: empty vector, OE: overexpression. (A) Western blotting results of synaptic plasticity‐related proteins. (B–E) Quantification of the expression level of TRKB, neuroligin, synaptophysin, and MBP in the brain in group sham, EV, and ANXA6 OE. *p < 0.05; **p < 0.01; ***p < 0.001.

Western blotting was conducted to detect the expression levels of synaptic plasticity–related proteins in brain tissue (Figure 4A). Figure 4B–E display the statistical results. The expression levels of TrkB (*p* = 0.002), MBP (*p* = 0.002), neuroligin (*p* = 0.002), and synaptophysin (*p* = 0.002) in the brains of the EV group were significantly lower than those in the sham group. Similarly, the expression levels of these proteins in the brains of the ANXA6 OE group were higher than those in the EV group, with statistically significant differences observed only in synaptophysin (*p* = 0.006) and MBP (*p* = 0.010).

#### 3.3.2 sh ANXA6 decreased the level of synaptic plasticity‐related proteins in the brain

**FIGURE 5:**
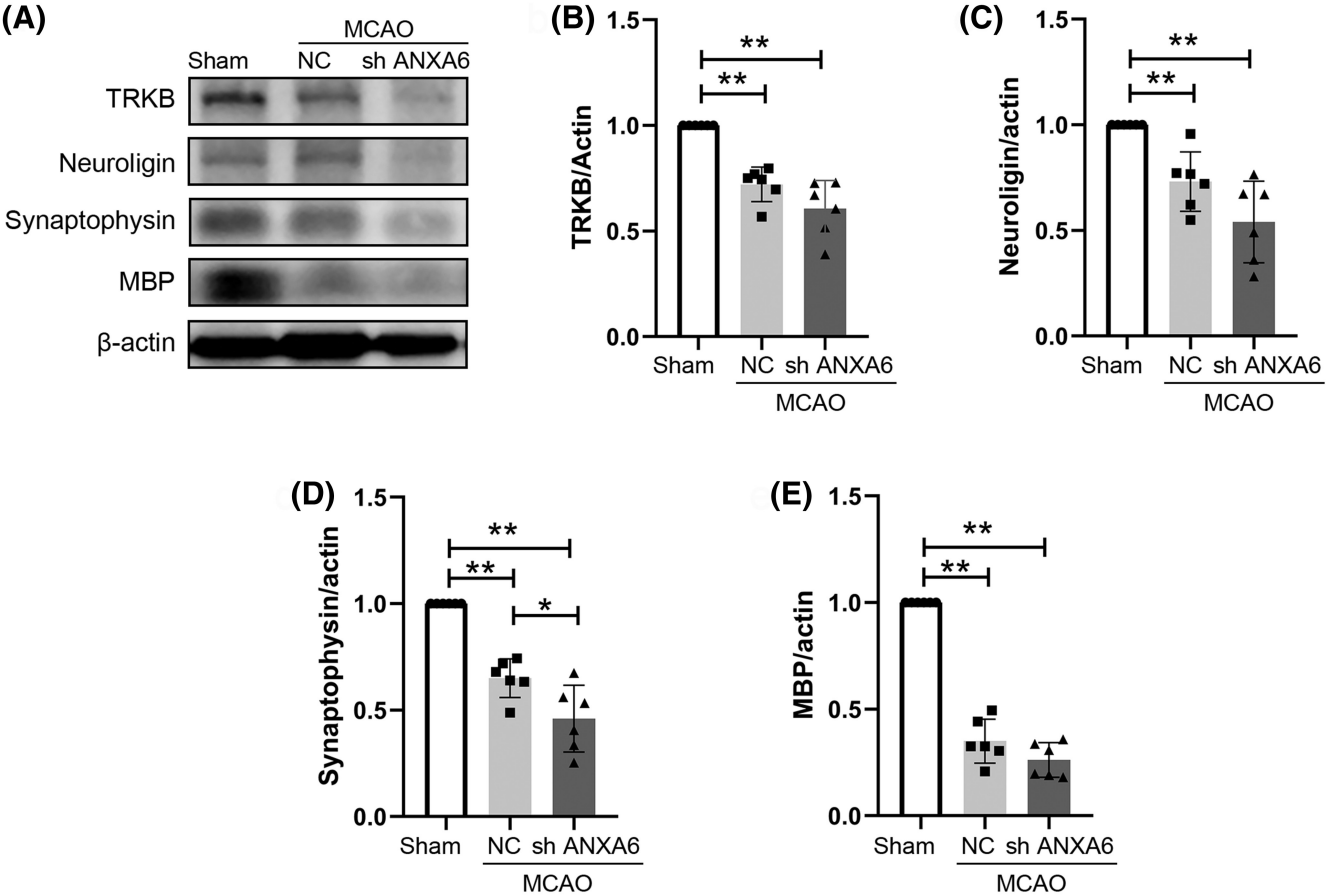
sh ANXA6 decreases the expression level of synaptic plasticity‐related proteins. MCAO: middle cerebral artery occlusion. TRKB: Tropomyosin‐related kinase B, MBP: Myelin basic protein. NC: nonsensical oligonucleotide control. (A) Western blotting results of synaptic plasticity‐related proteins. (B–E) Quantification of the expression level of TRKB, neuroligin, synaptophysin, and MBP in the brain in group sham, NC, and shANXA6. *p < 0.05; **p < 0.01; ***p < 0.001.

Figure 5A displays western blotting results for synaptic plasticity–related proteins in brain tissue, and Figure 5B–E display the statistical results. Compared to the sham group, the expression levels of TrkB (*p* = 0.002), MBP (*p* = 0.002), neuroligin (*p* = 0.002), and synaptophysin (*p* = 0.002) in the brains of the NC group were significantly decreased. The expression levels of these proteins in the brains of the sh ANXA6 group were lower than those in the NC group, with a statistically significant difference observed only in synaptophysin (*p* = 0.037).

### 3.4 Plasma ANXA6 levels and correlation with clinical characteristics

**FIGURE 6:**
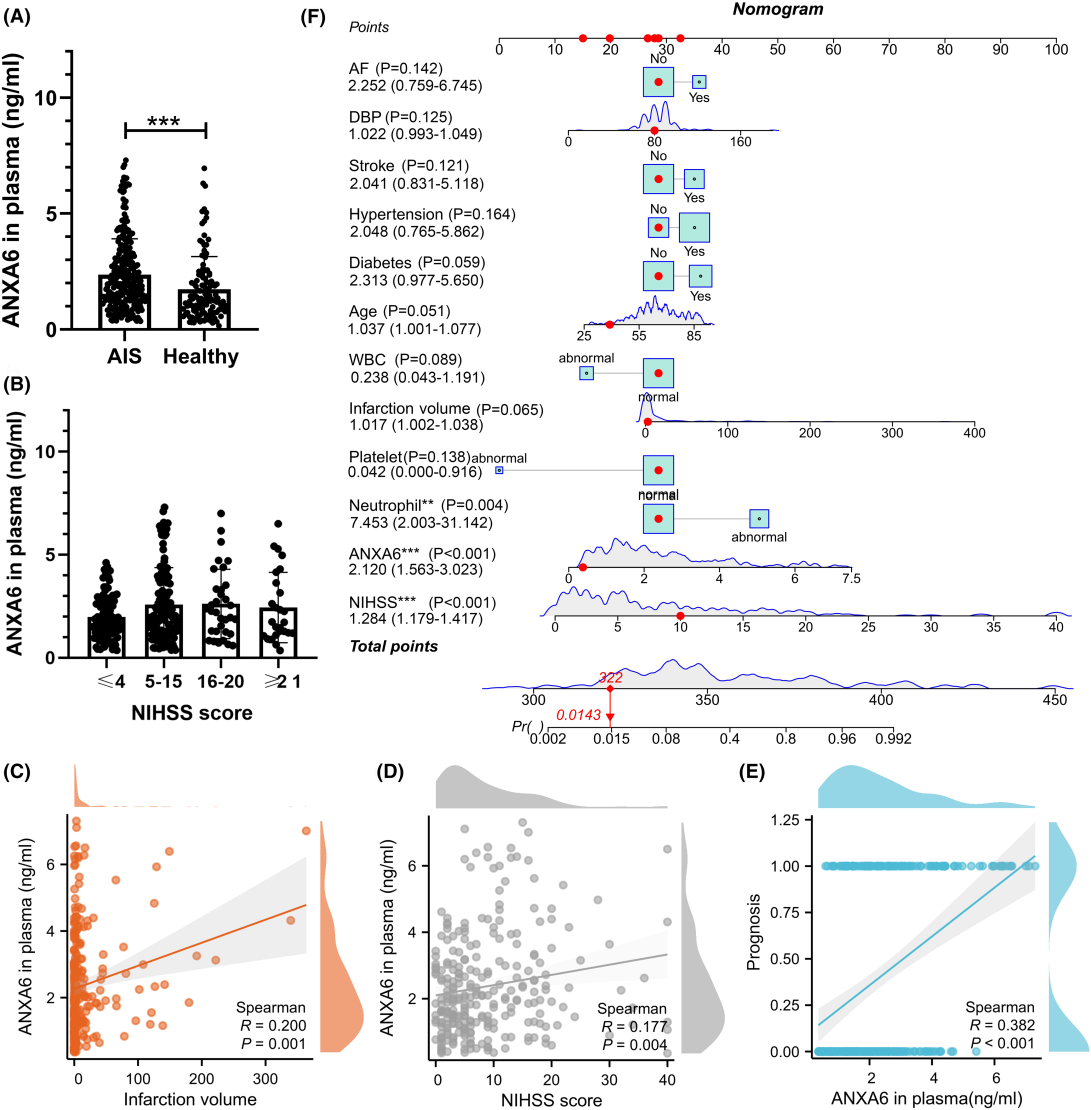
The clinical significance of plasma ANXA6 level in acute ischemia stroke. AIS: acute ischemic stroke, NIHSS: National Institute of Health Stroke Scale, AF: atrial fibrillation, DBP: diastolic blood pressure, WBC: white blood cell. We collected the plasma of healthy controls, and the patients experienced AIS within 24 h. (A): Plasma ANXA6 level of healthy controls and patients with AIS. (B) Plasma ANXA6 level of patients with AIS (grouped by NIHSS score). (C–E) Scatter plots display the Spearman Correlation test between ANXA6 level and infarction volume, NIHSS score, and prognosis. (F) The nomogram displays the logistic regression model results, identifying the independent risk factors of poor AIS outcomes. *p < 0.05; **p < 0.01; ***p < 0.001.

We included 268 patients with AIS and 120 healthy volunteers in this study, and their baseline information are reviewed in Table S2. We used ELISA to measure plasma ANXA6 levels in patients with AIS and healthy controls. Figure 6A shows plasma ANXA6 levels were significantly higher in patients with AIS than healthy controls (*p* < 0.001). In Figure 6B, plasma ANXA6 levels in the NIHSS (≤4) group are the lowest, with no statistical significance. However, ANXA6 levels in the other three groups showed no significant differences. In addition, Figure 6C–E show a slightly positive correlation between plasma ANXA6 levels and infarction volume (*r* = 0.200, *p* = 0.001), NIHSS score (*r* = 0.177, *p* = 0.004), and poor stroke outcomes (*r* = 0.382, *p* < 0.001).

### 3.5 Plasma ANXA6 levels serve as an independent risk factor for poor AIS prognosis

We established a logistic regression model. We identified ANXA6 as an independent risk factor for AIS outcomes (odds ratio [OR] = 2.120; 95% CI = 1.563–3.023; *p* < 0.001) (Figure 6F), indicating that each unit increase in ANXA6 is associated with 2.12 times higher likelihood of poor prognosis. Similarly, NIHSS was also identified as a predictor (OR = 1.284; 95% CI = 1.179–1.417; *p* < 0.001), indicating that each unit increase in NIHSS score was associated with 1.284 times higher likelihood of poor prognosis. Furthermore, an abnormal neutrophil count (OR = 7.453; 95% CI = 2.003–31.142; *p* < 0.001) was also identified, signifying that patients with abnormal neutrophil levels have a 7.453 times greater risk of experiencing a poor prognosis than those without.

## 4 DISCUSSION

Given the absence of existing literature on ANXA6 and cerebral ischemic stroke, we first explored the variation trends in ANXA6 expression levels in the brain and plasma at different periods after reperfusion. Our results showed that ANXA6 levels in the brain tissue of the mouse MCAO model were significantly decreased at 6 h and 1 day post I/R and then gradually increased to normal levels by Day 28. Interestingly, the variation in ANXA6 levels in the brain and plasma exhibited contrasting trends. The annexin family binds to electronegative phospholipids in a Ca^2+^‐dependent manner, participating in cell adhesion, migration, and membrane repair.^9^ ANXA6, observed in damaged neurons, forms a repair cap at the damage sites, aiding in the repair of neuron membranes.^9^ Moreover, ANXA6 accumulates in the axon initial segment during neuron development.^27^ Brain autopsies of patients with hypoxic–ischemic injuries have shown ANXA6 to be located adjacent to axons and cell membranes.^6^ However, no published literature exists on ANXA6 levels in the brain following focal ischemia. We concluded that focal ischemia induces cellular membrane damage, releasing ANXA6 into the plasma. According to the literature, ischemia can destroy cellular membrane integrity of neurons.^28^, ^29^ Cellular membrane damage allows for intracellular Ca^2+^ accumulation, disrupting ion gradients, increasing protease activity, and inducing mitochondrial dysfunction, ultimately resulting in neuron degeneration and apoptosis.^9^ Our results show that in the MCAO model, brain ANXA6 levels gradually increased to normal levels approximately 1 month after onset. Given the potential role of ANXA6 in membrane repair and axon sprouting, we deduced that increased ANXA6 levels in the brain could be attributed to the activation of spontaneous recovery mechanisms. Focal ischemia can trigger a series of reactions to promote spontaneous neural recovery. Recovery can be categorized into three stages: (1) several hours after onset (a possibility of repairing the impaired tissue), (2) several days to weeks after onset (initiation of spontaneous recovery), and (3) the chronic recovery stage (a relatively stable period of potential adjustments in cerebral function).^30^ In our study, ANXA6 levels in brain tissue gradually increased 3 days after onset. This timepoint may be called the second recovery stage (several days to weeks after onset), consistent with the initiation of spontaneous recovery mentioned in the literature.^30^ Therefore, we concluded that the gradual decrease in plasma ANXA6 levels corresponds with cellular membrane repair. In our study, plasma ANXA6 levels in the AIS group were significantly higher than those in the healthy controls, consistent with the results of the mouse MCAO model. Additionally, plasma ANXA6 levels positively correlated with infarction volume and NIHSS scores. Therefore, plasma ANXA6 levels may serve as biomarkers for identifying poor AIS outcomes. These results suggest that a larger infarction volume and a higher NIHSS score may indicate greater damage to brain tissue, more severe membrane damage, and increased plasma ANXA6 levels, indicating the potential of ANXA6 as an independent risk factor for AIS outcomes. Additionally, the mouse MCAO model results showed that ANXA6 OE significantly decreased infarction volume and improved neurological function, indicating the protective role of ANXA6 in AIS.

The ischemia‐induced destruction of the blood–brain barrier and cellular membrane are the primary causes of brain edema and neuron necrosis.^28^ After axon damage, cell membrane resealing is vital for neuron regeneration.^9^ Given that ANXA6 can significantly alleviate neurological deficits and participate in membrane repair following AIS, we explored whether ANXA6 can regulate neuroplasticity after AIS. Neuroplasticity refers to the capacity of the brain to adapt to its current microenvironment during growth and development. Even after a stroke, the brain tissue retains neuroplasticity.^31^ This poststroke neuroplasticity involves reconstructing neural networks and functional responses in neurogenic niches.^32^ Increased plasticity allows neurogenesis, including axon sprouting, synapse generation, and neurological function remapping.^31^ Neuroplasticity is vital for recovering cerebral function after injury.^33^ However, poststroke function recovery is extremely limited, causing long‐term neurological dysfunction. This limited functional recovery may be due to limited neurogenesis.^31^ Axon sprouting, synapse remodeling, and central microenvironment regulation have been identified as targets for alleviating post‐stroke neurological deficits.^31^

Synaptophysin belongs to the synaptic vesicle membrane protein family and is widely distributed in brain tissue. Synaptophysin is highly expressed during synaptogenesis, regulating synapse formation and synaptic vesicle cycling.^34^ Located on nervous terminals, it detects axonal sprouting and synaptogenesis during neural remodeling and development. Increased synaptophysin levels in cerebral tissue may indicate increased neuroplasticity following AIS.^33^ In our study, synaptophysin expression levels in brain tissue were significantly decreased at 7 days after AIS. This result aligns with a previous study demonstrating significantly down‐regulated synaptophysin levels in the hippocampus after focal cerebral ischemia.^35^ Furthermore, increased synaptophysin levels can improve working memory deficits.^36^ ANXA6 OE significantly increased synaptophysin levels in the brain compared to the EV group. Notably, this study is the first to report the interaction between ANXA6 and synaptophysin.

MBP, which participates in the mitogenicity of myelin‐enriched fractions,^37^ is typically located on the major dense line of multilamellar membranes (cytoplasmic side), maintaining the internal structure of oligodendrocyte membranes.^38^ MBP is crucial for myelin generation. MBP can bind with obvious polyanionic surfaces, such as lipid bilayers and several cytoskeleton proteins, participating in myelin adhesion and cellular signal transmission.^39^ In our study, focal ischemia significantly decreased MBP levels in the brain, consistent with previous studies that indicate that oxygen–glucose deprivation can disrupt MBP distribution, decreasing the interaction between neurons and MBP.^40^ MBP accumulates in the oligodendroglia–myelin complex, serving as a marker of demyelination.^41^ Additionally, cerebral ischemia may induce cell disintegration, releasing cell‐specific proteins into the cerebrospinal fluid (CSF).^42^ Increased MBP concentration in the CSF positively correlates with more severe brain injury. Elevated MBP levels in the CSF may indicate a relatively poor outcome.^43^ Our results suggest that ANXA6 OE increases MBP levels, potentially enhancing synaptic plasticity in patients with AIS. According to the existing literature, the remyelination of axons in the zone adjacent to the infarction lesion is crucial for remodeling neuronal transmission and neural function pathways.^44^

Neuroligins belong to the type I transmembrane protein family, containing a cytoplasmic C‐terminal domain that can modulate synaptic plasticity.^45^ Effective neuron transmission depends on the collaboration of neurotransmitter receptors, cell adhesion molecules, scaffolding proteins, and signaling proteins.^46^ As synapse adhesion molecules, neuroligins typically reside on the postsynaptic membrane, participating in the differentiation, maturation, and stabilization of synapses and regulating the connections between neurons and synaptic transmission.^47^, ^48^ Neuroligins can significantly increase the number of spines and synapses in neurons and are involved in initial synapse generation.^45^ The lateral interaction between neuroligins is vital for transmitting transsynaptic signals.^47^ In our study, the levels of neuroligins in the brain of the MCAO group were significantly decreased compared to the sham group. Consistent with the existing literature, neuroligin levels in cultured neurons were significantly decreased 8 h after oxygen–glucose deprivation.^48^ Our results suggest that ANXA6 OE may slightly increase neuroligin levels, with no statistical significance. Further studies are required to explore the interactions between ANXA6 and neuroligins.

TrkB is extensively distributed in the brain, typically accumulating on the periphery of synaptic membranes. Activating TrkB‐related signals can promote neurotransmitter release from presynaptic terminals.^49^ TrkB is involved in various biological processes, including synapse generation, transmission, plasticity, axon and dendrite sprouting, and nervous network remodeling.^49^, ^50^, ^51^ In our study, focal ischemia significantly decreased TrkB levels in the brain compared to the sham group. This finding aligns with a previous study showing that TrkB levels were significantly decreased in the brain tissue of a mouse MCAO model.^52^ Moreover, ANXA6 OE slightly increased TrkB levels with no statistical significance, and vice versa. Our results indicate that ANXA6 may play a protective role in AIS by upregulating TrkB expression. Similarly, a study has demonstrated that TrkB agonists can alleviate neurological deficits and decrease infarction volume in a rat MCAO model.^53^ Therefore, TrkB represents a potential therapeutic target for neuron regeneration and neuronal network remodeling post stroke.^49^ However, our study had some limitations. First, the sample size of clinical subjects was small. Second, we examined ANXA6 expression in the brain using lentivirus ICV, which cannot be used in clinical settings.

## 5 CONCLUSIONS

In conclusion, our study is the first to propose a protective role of ANXA6 in AIS. Our results showed that ANXA6 OE increased TrkB, MBP, neuroligin, and synaptophysin levels in the brain following AIS. Furthermore, it decreased the infarction area and alleviated neurological deficits. Additionally, plasma ANXA6 levels can serve as biomarkers for identifying poor AIS outcomes. Our results suggest a novel neuroprotective concept in AIS.

## AUTHOR CONTRIBUTIONS

WYL wrote the main manuscript text. YZH completed animal experiments. WRL and ZYM prepared the figures. HZP and FJF analyzed the data. YF and LP collected clinical data. LYM supervised the study and revised the manuscript.

## FUNDING INFORMATION

This project was supported by the National Natural Science Foundation of China (NO. 81971222) and Beijing Municipal Natural Science Foundation (NO. 7222083).

## CONFLICT OF INTEREST STATEMENT

The authors declare that they have no competing interests. Luo, Yumin is an Editorial Board member of CNS Neuroscience and Therapeutics and a corresponding author of this article.

## DECLARATION OF HELSINKI

Our study conforms to the Declaration of Helsinki.

## Supporting information


Table S1.

Table S2.

## Data Availability

Data are available upon reasonable request.
