# Supplementary material for: Annexin A6 mitigates neurological deficit in ischemia/reperfusion injury by promoting synaptic plasticity
Source: CNS Neurosci Ther. 2024 Feb 21;30(2):e14639. doi: 10.1111/cns.14639 (PMC10880127; doi:10.1111/cns.14639)
Supplement: Supplementary file 1 — Table S1. [file CNS-30-e14639-s001.docx]

Supplementary table 1. The table elucidating the grouping strategy

| **Experiment I (n=5)** | | | | | | | | |
| --- | --- | --- | --- | --- | --- | --- | --- | --- |
| Sham | MCAO | | | | | | | |
| sham | I/R (6h) | I/R (1d) | | I/R (3d) | | I/R (7d) | | I/R (28d) |
| **Experiment II (n=6)** | | | | | | | | |
| Sham | MCAO | | | | | | | |
| sham | shRNA-ANXA6 | | NC | | ANXA6 OE | | EV | |

MCAO: middle cerebral artery occlusion; I/R: ischemia/reperfusion; NC: nonsensical oligonucleotide control; OE: overexpression; EV: empty vector.
