# Supplementary material for: Annexin A6 mitigates neurological deficit in ischemia/reperfusion injury by promoting synaptic plasticity
Source: CNS Neurosci Ther. 2024 Feb 21;30(2):e14639. doi: 10.1111/cns.14639 (PMC10880127; doi:10.1111/cns.14639)
Supplement: Supplementary file 2 — Table S2. [file CNS-30-e14639-s002.docx]

Supplementary table 2. Baseline information of all the participants.

|  | **Patients with AIS (n=268)** | **Healthy controls (n=120)** | ***P* value** |
| --- | --- | --- | --- |
| Age | 64.43±13.576 | 63.24±16.505 | 0.458 |
| Gender: male, No. (%) | 184(68.7) | 75(62.5) | 0.234 |

AIS: acute ischemic stroke.
